# Mouse Cytomegalovirus infection overrules T regulatory cell suppression on natural killer cells

**DOI:** 10.1186/1743-422X-11-145

**Published:** 2014-08-09

**Authors:** Marc Lindenberg, Gulhas Solmaz, Franz Puttur, Tim Sparwasser

**Affiliations:** Institute of Infection Immunology, TWINCORE, Centre for Experimental and Clinical Infection Research; a joint venture between the Medical School Hannover (MHH) and the Helmholtz Centre for Infection Research (HZI), Feodor-Lynen-Strasse 7, 30625 Hannover, Germany

**Keywords:** Mouse cytomegalovirus, Tregs, NK cells

## Abstract

**Background:**

Cytomegalovirus establishes lifelong persistency in the host and leads to life threatening situations in immunocompromised patients. FoxP3^+^ T regulatory cells (Tregs) critically control and suppress innate and adaptive immune responses. However, their specific role during MCMV infection, especially pertaining to their interaction with NK cells, remains incompletely defined.

**Methods:**

To understand the contribution of Tregs on NK cell function during acute MCMV infection, we infected Treg depleted and undepleted DEREG mice with WT MCMV and examined Treg and NK cell frequency, number, activation and effector function *in vivo*.

**Results:**

Our results reveal an increased frequency of activated Tregs within the CD4^+^ T cell population shortly after MCMV infection. Specific depletion of Tregs in DEREG mice under homeostatic conditions leads to an increase in NK cell number as well as to a higher activation status of these cells as compared with non-depleted controls. Interestingly, upon infection this effect on NK cells is completely neutralized in terms of cell frequency, CD69 expression and functionality with respect to IFN-γ production. Furthermore, composition of the NK cell population with regard to Ly49H expression remains unchanged. In contrast, absence of Tregs still boosts the general T cell response upon infection to a level comparable to the enhanced activation seen in uninfected mice. CD4^+^ T cells especially benefit from Treg depletion exhibiting a two-fold increase of CD69^+^ cells 40 h and IFN-γ^+^ cells 7 days p.i. while, MCMV infection per se induces robust CD8^+^ T cell activation which is also further augmented in Treg-depleted mice. Nevertheless, the viral burden in the liver and spleen remain unaltered upon Treg ablation during the course of infection.

**Conclusions:**

Thus, MCMV infection abolishes Treg suppressing effects on NK cells whereas T cells benefit from their absence during acute infection. This study provides novel information in understanding the collaborative interaction between NK cells and Tregs during a viral infection and provides further knowledge that could be adopted in therapeutic setups to improve current treatment of organ transplant patients where modulation of Tregs is envisioned as a strategy to overcome transplant rejection.

**Electronic supplementary material:**

The online version of this article (doi:10.1186/1743-422X-11-145) contains supplementary material, which is available to authorized users.

## Introduction

Mouse cytomegalovirus (MCMV) belongs to the family of β-herpes viruses and shares many attributes with human cytomegalovirus (HCMV). This makes it an attractive tool to study CMV associated immune responses in an infection model to better characterize the CMV-host relationship *in vivo*. CMV reactivation and primary infection pose as a major health concern in transplantation medicine leading to life threatening consequences in immunocompromised patients. As a means of suppressing transplant rejections in patients, one novel proposed strategy has been to adoptively transfer *ex vivo* expanded FoxP3^+^ T regulatory cells (Tregs) [1]. In order to better understand their role in acute CMV infection, this study sets out to elucidate their interaction with NK cells and effector T cells using an MCMV mouse model. Natural Tregs are major players in suppressing the immune system and are therefore important for controlling the balance between activation and tolerance [2, 3]. The transcription factor FoxP3 is a specific regulatory gene that distinguishes Tregs from other cell types and is important for their suppressive function [4]. A frameshift mutation in the FoxP3 gene locus on the X-chromosome in Scurfy mice results in a lethal multi-organ inflammation caused by a massive proliferation of effector T cells [5]. Despite the fact that Tregs are crucial for maintenance of the immune homeostasis, they are also known to suppress the immune system in several diseased conditions like cancer [6] or in the context of infections for example induced by viruses [7–13]. In doing so, they dampen pathogen-specific innate or adaptive immune responses and impede pathogen clearance from the host in most infectious settings. Treg suppression spans a diverse cohort of immune cells including monocytes, dendritic cells (DCs), NK cells, NKT cells, CD4^+^ and CD8^+^ effector T cells [14, 15]. They conduct their suppression using an arsenal of mechanisms such as modulating the bioavailability of IL-2 [16, 17], production of certain cytokines like IL-10, IL-35, TGF-β and signaling molecules like cAMP [18], direct killing [19] or downregulating co-stimulatory molecules CD80/86 on DCs via CTLA-4 by trans-endocytosis [20] and thereby indirectly suppress T effector responses. During acute MCMV infection, NK cells predominantly confer resistance against MCMV-induced pathogenesis by recognizing the viral m157 glycoprotein on infected cells via the Ly49H receptor [21–23]. Thus, mouse strains exhibiting NK cells equipped with this receptor like C57BL/6 are far more resistant than strains lacking it like BALB/c. According to Dokun et al [24, 25], the NK response to MCMV constitutes three phases. The first phase consists of an unspecific proliferation of NK cells with no preferential expansion of the Ly49H^+^-MCMV specific subset, which is postulated to be mostly cytokine dependent, followed by an MCMV-specific expansion and subsequent outgrowth of Ly49H^+^ cells within the NK cell population. In contrast to other Ly49 receptors, Ly49H associates with immunoreceptor tyrosine-based activation motifs (ITAMs) on the adaptor molecules DAP10 and DAP12, which are responsible for inducing proliferation and activation [22, 26]. The final phase consists of a slow contraction of the total NK cell response and frequency until baseline levels are achieved [24, 27].

Studies carried out by Ghiringhelli *et al*., demonstrated that mutant Scurfy mice lacking the functional gene FoxP3 exhibited, in addition to highly activated T effector cells, a 10 fold greater NK cell proliferation [28]. Furthermore, enhanced cytotoxicity of NK cells was observed as compared with WT mice with no added influence on their activation state. *In vitro* studies as well as tumor mouse models provided evidence that a direct control of Tregs on NK cells may exist and results in impaired functionality of NK cells in the presence of Tregs [28–30]. Membrane-bound Transforming growth factor beta was proposed to be involved in this process, since blocking antibodies of this complex abolished the observed effects [28]. Recent studies by Gasteiger et al. showed an indirect interaction mediated by increased IL-2 levels produced by CD4^+^ T cells upon Treg depletion [31, 32]. IL-2 signaling on NK cells induced proliferation and additionally enhanced their cytotoxic function via increased sensitivity for target cells.

These observations led us to ask the question whether this interaction between NK cells and Tregs is also of importance in a viral model like MCMV, where NK cell proliferation is initially cytokine dependent and later driven by signaling of the NK cell-activating receptor Ly49H.

Here, we show that boosting effects of Treg depletion on NK cells under homeostatic conditions are overruled upon MCMV infection with no preferential effects on Ly49H subsets. The viral clearance remains unchanged even though we observe enhanced general T cell activation, highlighting the outstanding role of NK cells in controlling MCMV infection in C57BL/6 mice. These results clearly indicate that the role of Treg-mediated suppression on NK cells activated by MCMV infection is at best negligible, whereas the activation of T cells is further enhanced in the absence of Tregs.

## Results

### MCMV infection leads to elevated FoxP3^+^ Tregs in the CD4^+^ T cell compartment

**Figure 1:**
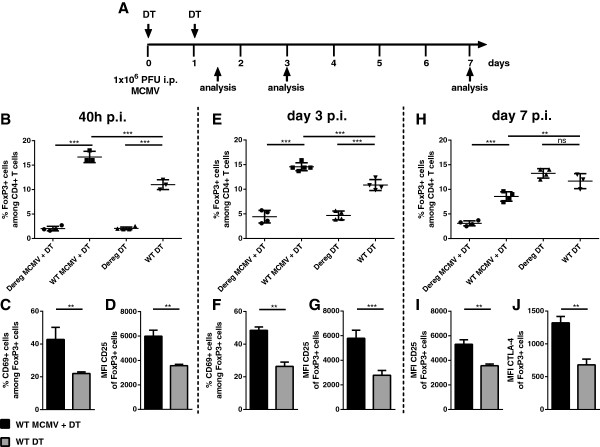
**MCMV infection elevates Treg proportion in CD4** ^**+**^ **T cell compartment early upon infection and DT administration results in efficient depletion of Tregs in DEREG mice. (A)** Infection and depletion scheme of experimental procedure. **(B)** FoxP3^+^ cells among splenic CD4^+^CD3^+^ cells 40 h p.i. **(C)** the proportion of CD69^+^ cells among them and **(D)** their mean fluorescence intensity (MFI) of CD25 expression. **(E)** shows the percentage of FoxP3^+^ cells in the CD4^+^ T cell compartment on day 3 p.i., **(F)** indicates the CD69^+^ cells within this subset and **(G)** the MFI of CD25 expression. **(H)** The frequency of FoxP3^+^ cells among CD4^+^ T cells on day 7 p.i. **(I)** shows the CD25 expression on FoxP3^+^ cells and **(J) the** MFI of CTLA-4 expression on FoxP3^+^ cells on day 7 p.i. Data shown are from one representative experiment out of three in case of frequency analysis **(B)**, **(E)** and **(H)** and out of at least two with regard to activation markers **(C)**, **(D)**, **(F)**, **(G)**, **(I)** and **(J)** using 3-5 mice per group. Significance of differences between means of groups was calculated by two tailed, unpaired Student’s t-test. (**) p < 0,01, (***) p < 0,001, (ns) not significantly different.

Cytomegalovirus has developed a number of immune-evasion mechanisms to prolong its survival within the host [33, 34]. As Tregs display certain features to be a possible target of immune evasion mechanisms, we set out to characterize in detail the effect of MCMV on Treg properties during the course of acute infection. We first examined the Treg response initiated by MCMV infection in the spleen as a site for primary MCMV replication. We observed a significant increase in the frequency of these cells among CD4^+^ T cells from 40 hours post infection (h p.i.) (Figure 1B) with a similar increase in the absolute number of Tregs (Additional file 1: Figure S1). This increase in Tregs persisted even at day 3 p.i. when compared with mock-infected mice (Figure 1E) and is irrespective of DT treatment (Additional file 1: Figure S1D). Furthermore, a larger proportion of infection-induced Tregs showed a higher activation state indicated by increased early activation marker CD69 expression after 40 h (Figure 1C) and 3 days p.i. (Figure 1F). This increase was evident even by the mean fluorescence intensity (MFI) of CD25 40 h p.i. (Figure 1D) and on day 3 p.i. (Figure 1G). On day 7 p.i., representing the peak phase of the T cell response to MCMV with regard to non-inflationary T cell epitopes [35, 36], FoxP3^+^ cells were significantly reduced amongst the CD4^+^ T cell population (Figure 1H) but still showed an increased MFI of CD25 (Figure 1I) and of CTLA-4 (Figure 1J). Hence, we hypothesized that depletion of FoxP3^+^ cells may result in an enhanced anti-viral immune response. To investigate the influence of Tregs during the acute phase of the infection, we used DEREG mice, allowing for selective depletion of FoxP3^+^ Tregs by Diphtheria toxin (DT) administration [5]. Our data demonstrates that DT treatment on day 0 and day 1 p.i. (Figure 1A) results in efficient depletion of Tregs in our infection model at all time points of analysis (Figure 1B, E and H). The efficiency of the depletion is depicted in Additional file 2: Figure S2B and is also represented in the total number of Tregs (Additional file 1: Figure S1A, B and C). Even though Treg frequencies reached WT levels by day 7 after the first DT injection under homeostatic conditions, they remained significantly lower in infected Treg-depleted mice (Figure 1H and Additional file 2: Figure S2B). Hence, we determined that DEREG mice serve as an efficient tool for investigating acute MCMV disease progression in the absence of Tregs.

### Depletion of Tregs enhances NK cell frequency, number and activation state under homeostatic conditions with no added influence upon MCMV infection

**Figure 2:**
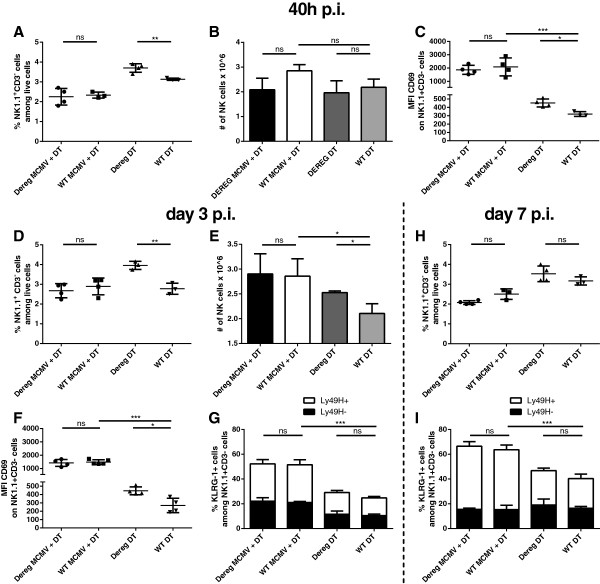
**The Treg depletion associated boosting effect on NK cells under homeostatic conditions is neutralized upon MCMV infection. (A)** Frequency of NK cells and **(B)** number of NK cells gated on NK1.1^+^CD3^-^ cells among live splenocytes and **(C)** their expression of CD69 as MFI 40 h p.i. **(D)** Proportion and **(E)** absolute number of splenic NK cells 3 days p.i. **(F)** MFI of activation marker CD69 and **(G)** maturation marker KLRG-1^+^ cells, stratified according to Ly49H expression, on day 3 p.i. **(H)** NK cells among live cells on day 7 p.i and **(I)** their expression of KLRG-1 again stratified according to Ly49H expression. Data shown are from one representative experiment out of three in case of day 3 p.i. analysis **(D)**, **(E)**, **(F)** and **(G)** and out of at least two with regard to 40 h and 7 days p.i. **(A)**, **(B)**, **(C)**, **(H)** and **(I)** using 3-5 mice per group. Significance of differences between means of groups was calculated by two tailed, unpaired Student’s t-test. (*) p < 0,05, (**) p < 0,01, (***) p < 0,001, (ns) not significantly different.

NK cells are important cellular mediators of the immune response needed to control MCMV infection. Previous studies have demonstrated that Scurfy mice bear functionally impaired Tregs [37] and therefore show higher numbers of activated NK cells [28]. To better elucidate the relationship between Tregs and NK cells, we investigated the effect of Treg depletion on NK cells during acute MCMV infection. We found that under homeostatic conditions, DEREG mice that were depleted of Tregs showed significantly higher frequencies of NK cells with comparable NK cell number after 40 h p.i. (Figure 2A and B) but this effect of depletion on NK cells was even more pronounced at day 3 p.i. (Figure 2D) and was reflected in the frequency and the total number of NK cells per spleen at this time point (Figure 2E). The increase in NK cells correlated with Treg depletion as on day 7 p.i., when Tregs reached wild type levels in mock infected mice, no differences in NK cell frequency and number were detectable between the two groups (Figure 2H). Surprisingly, the NK cell boosting effect of Treg depletion is completely abolished upon MCMV infection. No increase in NK cell frequency was observed in infected mice (Figure 2A and D), while activation state assessed by CD69 expression (Figure 2C and F) or maturation determined by KLRG-1 expression (Figure 2G and I) did not differ in the whole NK cell population as well as in the Ly49H^+^ NK cell compartment (Figure 2I and Additional file 3: Figure S3A, B). Furthermore, infection failed to alter the frequency and number of Ly49H^+^ NK cells even at a late time-point of day 7 p.i.. Although we detected a notable increase in CD69 expression upon DT treatment of DEREG mice, under homeostatic conditions after 40 h, 3 days and 7 days p.i. (Figure 2C, F and Additional file 3: Figure S3B), analysis of the maturation state of Ly49H^+^ versus Ly49H^-^ NK cells revealed an unaltered composition of MCMV-specific versus unspecific NK cells in uninfected as well as infected mice (Figure 2G and I) although infection increased KLRG1 expression irrespective of Treg depletion compared to uninfected mice (Infected ≥ 60% of NK cells versus uninfected ≤40% of NK cells) (Figure 2I). These findings suggest that Treg depletion fails to favor an outgrowth of either of the two NK cell subsets neither is the maturation altered. Hence, simultaneous ablation of Tregs and MCMV infection does not enhance the number or alter the phenotype of NK cells in contrast to steady state depletion.

### Interferon-γ production of NK cells in response to MCMV is not further enhanced in the absence of Tregs

**Figure 3:**
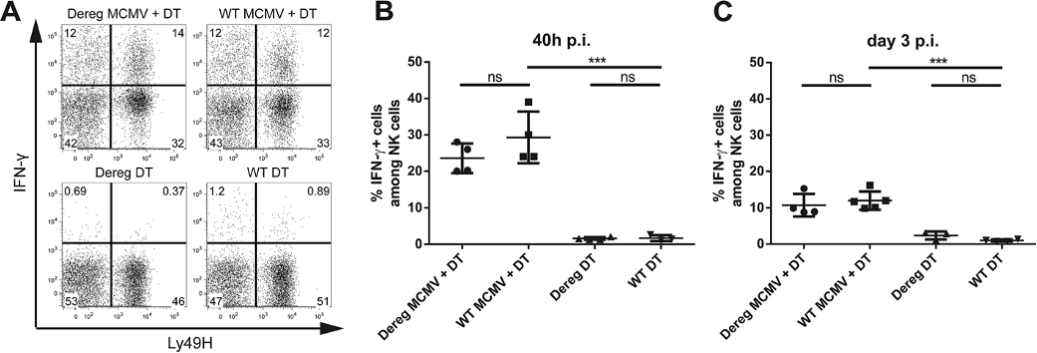
**IFN-γ** **expression of NK cells upon infection remains unchanged after Treg depletion. (A)** Representative FACS plots showing the IFN-γ expression of live NK1.1^+^CD3^-^ cells after IL-2 *ex vivo* stimulation and surface expression of Ly49H. **(B)** and **(C)** quantification of IFN-γ^+^ NK cells 40 h and 3 days p.i. Data are representative of two **(B)** or three **(C)** individual experiments with 3-5 mice per group. Significance of differences between means of groups was calculated by two tailed, unpaired Student’s t-test. (***) p < 0,001, (ns) not significantly different.

During the acute phase of MCMV infection Interferon-γ (IFN-γ) proves indispensable for effective MCMV control with NK cells being the main producers early after infection. Aside from granzymes and perforin, IFN-γ production constitutes one of the most important counteractive measures by NK cells against viral propagation [38–41]. Therefore, to test for functional consequences of Treg depletion on NK cells, we performed intracellular FACS staining of IFN-γ after a 4-hour restimulation with IL-2 in the presence of Brefeldin A. Using this protocol, modified from Mitrovic et al. [42], we could demonstrate that about 25% of NK cells from infected animals expressed IFN-γ (Figure 3A and B) as compared with mock- infected WT DT-treated animals after 40 h p.i. which showed a minor unspecific proportion of IFN-γ^+^ NK cells accounting for ≈ 2% of NK cells. However, quantification of the frequency of the IFN-γ^+^ NK cells revealed no differences between DEREG MCMV + DT-treated and WT MCMV + DT-treated mice, neither at the peak phase of IFN-γ production of NK cells, at 40 h p.i., nor at day 3 p.i. (Figure 3B and C). Fogel et al. reported a correlation between CD69 expression and IFN-γ production of NK cells [43]. Interestingly, although we observed a minor increase in CD69 expression on NK cells at these time points upon Treg depletion under naïve conditions, the increase in activation did not reflect the ability to produce IFN-γ as DEREG DT-treated mice showed comparably low frequencies of IFN-γ^+^ NK cells as WT DT-treated mice. Analysis of the Ly49H sub-compartments again showed no preferential effect of Treg depletion on either population (Figure 3A and data not shown). To rule out any biasing effects of IL-2 *ex vivo* restimulation, we performed additionally a PMA/Ionomycin restimulation assay on NK cells which revealed the same results but showed higher activation in general (Additional file 3: Figure S3C).

### Viral burden remains unaltered upon ablation of Tregs

**Figure 4:**
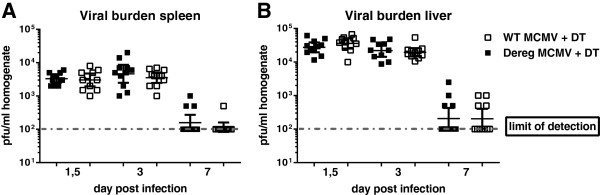
**Treg depletion has no effect on viral clearance in spleen and liver of C57BL/6 DEREG mice. (A)** Plaques developed after inoculation of sub-confluent mouse embryonic fibroblast (MEF) layers with spleen homogenates of infected mice obtained 40 h, 3 days and 7 days p.i. **(B)** Viral burden of the liver at the indicated time points. Data depicted shows geometric mean with 95% confidence interval of three pooled experiments with 3-5 mice per group. Limit of detection was determined by cell toxicity of low diluted homogenates for MEFs.

In order to examine the contribution of Treg depletion to viral clearance, we measured the viral load in the infected-depleted mice versus infected-non-depleted ones at different days p.i. Our results showed equally high viral loads in spleen and liver from both experimental mouse groups over the course of infection (Figure 4A and B). On day 7 p.i., viral burden was close to the detection limit in these organs and not detectable in the salivary glands (data not shown) of DEREG MCMV + DT-treated mice as well as WT MCMV + DT-treated ones, with no added differences upon Treg depletion. Overall, we show that viral clearance in immunocompetent DEREG mice, on C57BL/6 genetic background, is independent of Treg mediated function.

### Ablation of Tregs results in a boosted general T cell response

**Figure 5:**
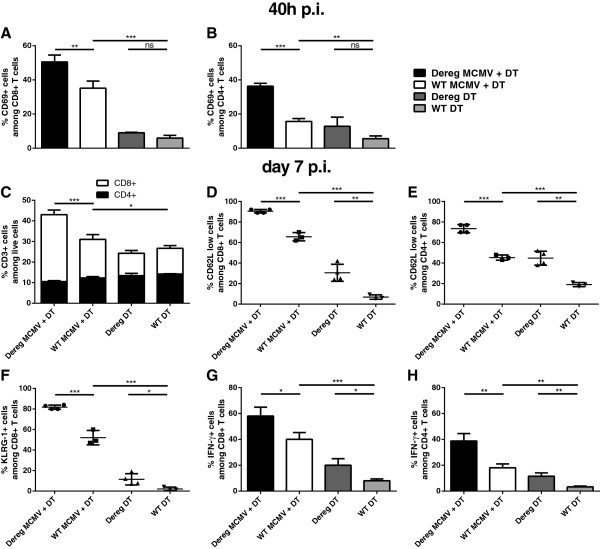
**Absence of Tregs enhances adaptive immune response of CD4** ^**+**^ **and CD8** ^**+**^ **T cells. (A)** Proportion of CD69^+^ among CD8^+^ and **(B)** CD4^+^ T cells 40 h p.i. **(C)** Percentage of CD3^+^ cells among live splenocytes on day 7 p.i. stratified by CD8 and CD4 expression. **(D)** CD62L^low^ cells within the CD8^+^ and **(E)** CD4^+^ T cell compartment as well as **(F)** KLRG-1 expression of CD8^+^ T cells 7 days p.i. **(G)** Quantification of IFN-γ^+^ cells among CD8^+^ T cells and **(H)** CD4^+^ T cells upon *ex vivo* stimulation with PMA / Ionomycin obtained from spleens of mice infected for 7 days. Data shown are from one representative experiment out of three for **(A)**, **(B)**, **(C)**, **(D)**, **(E)** and out of two **(F)**, **(G)** and **(H)** using 3-5 mice per group. Significance of differences between means of groups was calculated by two tailed, unpaired Student’s t-test. (*) p < 0,05 (**) p < 0,01, (***) p < 0,001, (ns) not significantly different.

The unaltered viral load in spleen and liver raised questions about the influence of Treg depletion on the adaptive T cell response upon MCMV infection and its impact on viral clearance. DEREG MCMV + DT-treated mice showed an early and significant increase in activated T cells assessed by CD69 expression in both the CD8^+^ as well as more pronounced, in the CD4^+^ compartment after 40 h p.i. as compared with WT MCMV + DT-treated mice (Figure 5A and B). Since day 7 p.i., represents the peak phase of T cell expansion and activation with regard to non-inflationary T cell epitopes upon MCMV infection, we examined the influence of Treg depletion at this time point and observed that general T cell responses are indeed enhanced. Overall, the frequency of T cells among splenic cells is increased with a simultaneous and significant increase in the ratio of CD8^+^ to CD4^+^ T cells in infected, Treg-depleted mice (Figure 5C). Furthermore up to 90% of CD8^+^ T cells and 70% of CD4^+^ T cells expressed low mean fluorescence intensity for CD62L as compared with 65% and 45% respectively in WT MCMV infected animals (Figure 5D and E). Strikingly, MCMV infection induced KLRG-1 expression in half of all CD8^+^ T cells, while infection plus Treg depletion further enhanced the maturation indicated by an increase of up to 80% of KLRG-1^+^ cells among CD8^+^ T cells (Figure 5F). In contrast to the influence on NK cells, the absence of Tregs also leads to higher frequencies of T cells responding with enhanced IFN-γ production in response to *ex vivo* restimulation (Figure 5G and H). Furthermore, the frequency of IFN-γ^+^CD4^+^ T cells increased two-fold in DEREG mice that were infected and DT treated. Overall, we show that Treg depletion and infection strongly promotes the expansion, activation and maturation of effector CD4^+^ and CD8^+^ T cells with simultaneous increases in IFN-γ production by both subsets.

## Discussion

CMV is a medically important DNA virus with high pathogenesis in immunocompromised and newborn individuals, representing a major reason for organ rejection in transplanted patients. Although anti-viral therapies to treat CMV disease are employed in the clinics, treatment is associated with poor oral bioavailability, development of anti-viral drug resistance over time and anti-viral drug related cytotoxicities [44]. Hence, there remains an urgent need to develop new anti-CMV compounds with different mechanism of action to reduce morbidity and contain infection. Thus, targeting Tregs has been suggested as a potential cell mediated approach for immunotherapy against infections [2].

A number of studies examining this concept, demonstrated the contribution of Tregs in promoting suppression of pathogen specific effector responses [7–10, 45–48], while others showed beneficial effects of Tregs upon infections [12, 49–52]. In this study, we set out to primarily examine the role of Tregs in modulating the MCMV-specific NK cell responses during the acute phase of infection, which until now remained incompletely defined. We observed elevated Treg frequencies among CD4^+^ T cells in the spleen early upon infection, indicating that MCMV infection may preferentially support differentiation of naïve T cells into Tregs, similarly described in a hepatitis virus infection model [53] where TGF-β induced by infection controlled this phenotypic change. To specifically address the question of whether the increase in Tregs influences the ongoing activation of innate and adaptive immune responses, we utilized DEREG mice to facilitate specific Treg depletion by Diphtheria toxin (DT) administration [5]. The advantage of the rapid and efficient depletion of Tregs in our model provided us the opportunity to infect mice on the day of first DT injection to really assess the influence of Tregs on NK cells during virus replication and thus minimize effects occurring prior to the onset of infection. This fact could account for the contrasting results Sungur et al. reported in terms of enhanced viral clearance upon CD25 antibody-mediated Treg depletion starting 2 days before infection [54]. In respect to these findings, we observed that under homeostatic conditions, depletion of Tregs significantly increased NK cell numbers and NK cell CD69 expression. Thus, depletion prior to infection could contribute to this discrepancy between both studies by conferring enhanced anti-viral defense already before infection. As Tregs return to baseline levels by day 7 p.i. in uninfected mice, our experimental mouse model avoids the development of artificial autoimmunity [55] and hence provides an unbiased approach to examine the phenotypes observed here upon infection. To further elucidate the interaction of Tregs with NK cells, and its influence on control of MCMV replication in C57BL/6 mice, we examined NK cell numbers and activation in the absence of Tregs. We detected elevated NK cell frequencies in uninfected DEREG mice depleted of Tregs consistent with findings reported in Scurfy mice and FoxP3 DTR knock-in mice [28, 32]. These cells additionally showed notably higher CD69 expression. In contrast, upon infection, we observed comparable NK cell responses between Treg-depleted and non-depleted mice. Studies by Fulton et al. and Lee et al. reported concordantly increased NK cell numbers in the lungs of Respiratory Syncytial Virus infected-BALB/c mice upon Treg depletion, which was carried out again by CD25 antibody administration starting already 3 days prior to infection [56, 57]. Using uninfected FoxP3 DTR knock-in mice, Gasteiger et al. pointed out that the increase of NK cell numbers upon Treg depletion corresponds to elevated CD127^+^ NK cell frequencies, expressing higher amounts of high affinity IL-2 receptor CD25 [31]. Therefore, enhanced IL-2 production by effector CD4^+^ T cells in the absence of Tregs may represent the likely mechanism underlying this phenomenon. This hypothesis was further substantiated by experiments that showed abrogation of this effect by blocking the IL-2 pathway or depleting the CD4^+^ T cell compartment [32] and similarly reported by Sitrin et al. in an autoimmune diabetes mouse model [58]. Our results in uninfected mice corroborate these findings as we similarly detected higher activation of CD4^+^ T cells upon Treg depletion. Although we observed a boosted CD4^+^ as well as CD8^+^ T cell response in DEREG MCMV + DT-treated mice as compared with WT MCMV + DT-treated mice, we could not detect differences in NK cell frequencies in infected mice suggesting that this proposed mechanism will need further clarification under a more infectious setting such as a salivary gland infection, where the demand for Ly49H^+^ NK cells would be further exemplified. A possible reason for this discrepancy could be that NK cells already achieve maximal proliferation upon tissue cultured MCMV infection and thus, fail to benefit from Treg depletion or elevated IL-2 levels. Treg depletion in MCMV-infected mice leads to higher proliferation of effector T cells, predominantly CD8^+^ T cells which represent the majority of T cells at day 7 p.i. Thus, consumption of IL-2 by proliferating CD8^+^ T cells which is not seen upon Treg depletion under homeostatic conditions, may offer another potential explanation. Treg ablation leads to similar frequencies of CD62^low^ CD4^+^ T cells as compared with those induced by MCMV infection alone. However, CD8^+^ T cells are significantly more activated upon MCMV infection than upon Treg depletion of naive mice and thus could abrogate IL-2 mediated effects. The insensitivity of the viral clearance to a boosted T cell response highlighted the importance of NK cells in limiting WT MCMV replication in C57BL/6 mice emphasized by a rapid clearance until day 7. The implications of Treg control over effector CD8^+^ T cell response would prove critical if the Ly49H receptor engagement was somehow abrogated as observed in the case of mice challenged with Δ*m157*-strain of MCMV, where CD8^+^ effector T cells critically governed the outcome of viral replication in infected organs [42]. In Ly49H^+^ NK cell competent C57BL/6 mice, we observed an initial viral burden that was already 100-fold reduced and close to the detection limit when the T cell response peaked. Our findings provide further support to the multi-functional importance of NK cells spanning the innate and adaptive arms of the immune system [59–61]. Furthermore, since MCMV infection primarily induces stronger CD8^+^ T cell responses, the contribution of the enhanced CD4^+^ T cell activation we observe upon infection in Treg depleted mice would require further investigation. CD4^+^ T cells are key players in establishing immunological memory and moreover known to develop cytotoxic abilities to directly attack infected cells under certain circumstances [62–64]. This makes them an important factor during MCMV infection and their importance may be further enhanced upon their suppression by Tregs. Thus, our results provide new evidence that Tregs play a role in modulating the immune response to MCMV infection, but this effect seems to be restricted to the suppression of adaptive immune cell activation. Our results suggest that Tregs enhance the general effector T cell response while NK cell function remains unaltered. This expansion in the CD8 T cell pool would warrant for further investigation into the contribution of Treg depletion on the antigen-specific effector T cell compartment after infection. The importance of Treg regulation on CD8 T cells in the absence of Ly49H-NK cell recognition has been recently analyzed in an independent study described by us in collaboration with Hansen and colleagues, showing enhanced activation, cytotoxicity and improved viral clearance in DEREG Balb/c mice depleted of Tregs [65]. Thereby, suggesting an important regulatory role by which NK-Ly49H function in concert with Tregs modulate anti-MCMV T cell effector responses [65]. This could be further extended into infection models in C57BL/6 mice employing a Δm157 MCMV strain, where the requirement for antigen specific T cells in viral clearance is further exemplified. Overall, our findings provide a foundation for the development of future Treg-mediated therapeutics in viral infections and in a broader context, in Treg-modulating strategies to overcome transplant rejection.

## Materials and methods

### Mice

Previously described DEREG mice on C57BL/6 background were used, allowing for the efficient and selective depletion of FoxP3^+^ T regulatory cells by the administration of Diphtheria toxin (DT) [5]. DT was administered in the amount of 25 ng/g bodyweight on both the day of infection and the following one. Male DEREG mice aged 8-12 weeks were used for experiments and gender- and aged-matched WT littermates served as controls. Mice were housed under specific pathogen free conditions at the animal facility of Twincore (Hannover, Germany). The protocol for this research study involving mice was approved by a suitably constituted Ethics Committee of the institution and was performed in accordance with animal welfare guidelines approved by institutional, state, and federal committees. Mice were sacrificed by CO_2_ asphyxiation in accordance with German animal welfare law. Every effort was made to minimize animal suffering.

### Virus

For infection, BAC derived MCMV WT Smith strain was used [66], which was kindly provided by Martin Messerle (Institute of Virology, Hannover Medical School, Germany). Virus propagation was carried out on doxycycline induced murine embryonic fibroblasts, also kindly provided by Dr. Tobias May from the Helmholtz Center for Infection Research and InSCREENeX (Braunschweig, Germany) [67]. Mice were infected with 10^6^ pfu of tissue cultured-derived virus by the intraperitoneal route.

### Plaque assay

Viral titers were determined by plaque assay performed on mouse embryonic fibroblasts (MEFs) as previously described [68]. Spleens and livers were frozen with 0.5 ml DMEM medium and after brief thawing homogenized using a TissueLyserLT (Qiagen) (50 Hz, 2:30 min). Ten-fold dilutions were prepared in duplicates and sub-confluent MEF layers were inoculated with homogenates for 2 h at 37°C. Following incubation, the inoculum was removed and cells were overlayed with 0.75% (w/v) carboxymethylcellulose (Sigma) in growth medium for each well. Plaques were counted after 6-8 days.

### Flow Cytometry

Red blood cells in single-cell suspensions of spleens were lysed using RBC lysis buffer (150 mM NH4Cl, 10 mM KHCO3, 0.1 mM EDTA). The isolated cells were counted by Trypan Blue exclusion and adjusted to the same cell number for FACS staining. After washing with PBS, cells were stained with the LIVE/DEAD^®^ Fixable Aqua Dead Cell Stain Kit (Invitrogen, Life Technologies GmbH, Darmstadt, Germany) to exclude dead cells. Following incubation with FACS buffer (0.25% BSA/ 2 mM EDTA in PBS) containing Fc-block (CD16/32, 2.4G2) for 10 min on ice cells were stained for surface markers with the following fluorochrome conjugated anti-mouse antibodies for 20 to 30 min on ice:

CD3 (145-2C11), CD4 (GK1.5), CD8α (53-6.7), CD25 (PC61.5), CD62L (MEL-14), CD69 (H1.2 F3), KLRG-1 (2 F1), Ly49H (3D10), NK1.1 (PK136).

Cells were fixed by using the Foxp3/Transcription Factor Staining Buffer Set (eBioscience, affymetrix, Frankfurt, Germany). Anti-mouse FoxP3 antibody FJK-16 s and anti-mouse CTLA-4 antibody UC10-4B9 (BioLegend, London, United Kingdom) were used for intracellular staining.

Unless otherwise stated, all antibodies were purchased from eBioscience, affymetrix (Frankfurt, Germany). Samples acquisition was performed on an LSRII Flow cytometer (BD Bioscience GmbH, Heidelberg, Germany), with the results analyzed using FlowJo software (Tree Star, Inc. Ashland, USA). Accurate gating was confirmed by single stains and fluorescence minus one controls, with non-specific binding was estimated by isotype controls. Cellular aggregates were excluded by SSC-W.

### *Ex vivo*stimulation assays

NK cell production of Interferon- γ (IFN-γ) was assessed by IL-2 re-stimulation in a 96-well U bottom plate. Splenocytes in the amount of 3×10^6^ were incubated with 250 U/ml IL-2 for 2 h initially, followed by an additional 2 h in the presence of 3 μg/ml BrefeldinA with 125 U/ml. For T cell *ex vivo* stimulation 25 ng/ml Phorbol-12-myristate-13-acetate (PMA) and 250 ng/ml Ionomycin were used for 4 h in the presence of 3 μg/ml BrefeldinA. Cells were stained for surface markers as described under Flow Cytometry. Intracellular staining for IFN-γ was performed after fixation in 2% PFA in PBS for 20 min on ice and permeabilization in PBS containing 0.25% BSA, 2 mM EDTA, and 0.5% saponin. PE conjugated anti-mouse IFN-γ antibody clone XMG1.2 (eBioscience, affymetrix, Frankfurt, Germany) was used.

### Statistics

Two-tailed, unpaired Student’s t-test was used to calculate the statistical significance of differences between means of groups or samples. A p-value <0.05 was considered significant, as indicated by asterisk signs: (*) for P < 0.05, (**) for P < 0.01 and (***) for P < 0.001.

## Electronic supplementary material

Additional file 1: Figure S1: MCMV infection induces increase in Treg frequency and number in infected Treg depleted mice. Total number of FoxP3^+^CD4^+^ Tregs 40 h (A), 3 days (B) and 7 days p.i. (C) and DT treatment. Infected wt mice showed increased Treg frequencies irrespective of DT treatment (D). Data shown is representative of at least 2 experiments, whereas data on day 3 p.i. is pooled from two individual experiments out of three, significance was determined by two tailed, unpaired Student’s t test. (*) p < 0,05. (PDF 14 KB)

Additional file 2: Figure S2: Selective depletion of Tregs in DEREG mice. (A) Gating Strategy for examining Treg frequencies. (B) Representative FACS plots showing the efficiency of Treg depletion in DEREG mice at 40 h, day 3 and day 7 p.i. Also included is a representative FACS plot showing undepleted control mice. Data shown is representative of two experiments with 2-4 mice per group. (PDF 408 KB)

Additional file 3: Figure S3: Activation and IFN-γ production by NK-Ly49H^+^ cells in Treg depleted mice. (A) Shows activation with regard to CD69 expression on Ly49H^+^NK1.1^+^ cells upon infection during Treg depletion. Data shown is representative of at least two experiments with 3-4 mice per group. (B) Expression of early activation marker CD69 on NK cells of infected mice is clearly reduced on day 7 in comparison to 40 h and 3 days p.i. But the results support the observation that Treg depletion under homeostatic conditions leads to higher NK cell activation. Data shown is representative of two experiments with 2-4 mice per group. (C) PMA/Ionomycin restimulation assay for splenic NK cell IFN-γ production showed similar results as IL-2 restimulation but with higher unspecific ex vivo activation. Groups consisted of 3-5 mice and significance was determined by two tailed, unpaired Student’s t test. (***) p < 0,0001; (ns) not significantly different. (PDF 21 KB)

## Abbreviations

DT: Diphtheria toxin
FoxP3: Forkhead-box-protein P3
KLRG-1: Killer cell lectin-like receptor G1
p.i.: Post infection
Treg: FoxP3^+^ T regulatory cell.
