# Supplementary figures and images for: Mouse Cytomegalovirus infection overrules T regulatory cell suppression on natural killer cells
Source: Virol J. 2014 Aug 9;11:145. doi: 10.1186/1743-422X-11-145 (PMC4254395; doi:10.1186/1743-422X-11-145)

Supplementary Figure 1

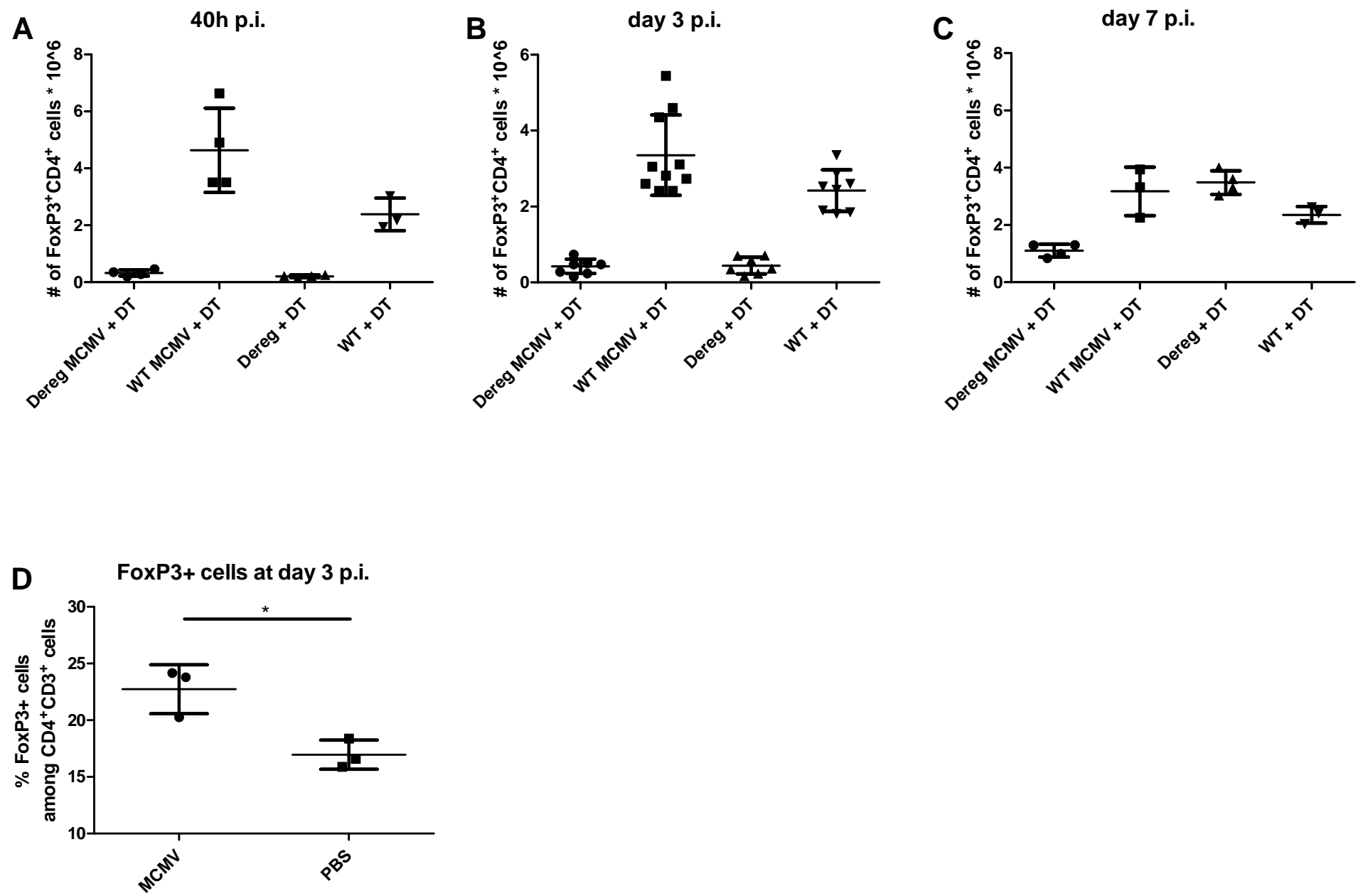

Supplement: Supplementary file 1 — Additional file 1: Figure S1: MCMV infection induces increase in Treg frequency and number in infected Treg depleted mice. Total number of FoxP3+CD4+ Tregs 40 h (A), 3 days (B) and 7 days p.i. (C) and DT treatment. Infected wt mice showed increased Treg frequencies irrespective of DT treatment (D). Data shown is representative of at least 2 experiments, whereas data on day 3 p.i. is pooled from two individual experiments out of three, significance was determined by two tailed, unpaired Student’s t test. (*) p < 0,05. (PDF 14 KB) [file 12985_2014_2471_MOESM1_ESM.pdf]

Supplementary figure 2

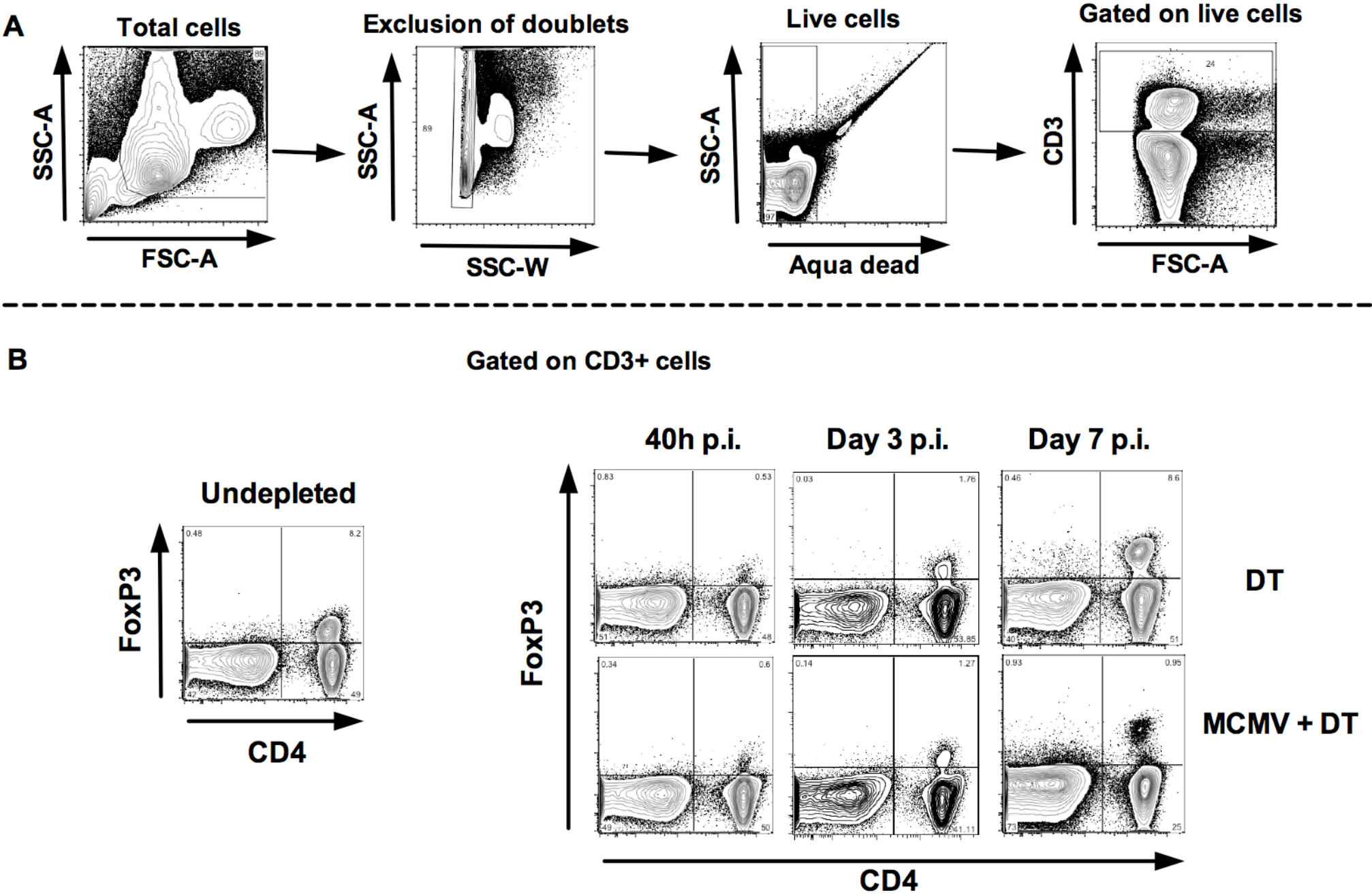

Supplement: Supplementary file 2 — Additional file 2: Figure S2: Selective depletion of Tregs in DEREG mice. (A) Gating Strategy for examining Treg frequencies. (B) Representative FACS plots showing the efficiency of Treg depletion in DEREG mice at 40 h, day 3 and day 7 p.i. Also included is a representative FACS plot showing undepleted control mice. Data shown is representative of two experiments with 2-4 mice per group. (PDF 408 KB) [file 12985_2014_2471_MOESM2_ESM.pdf]

Supplementary Figure 3

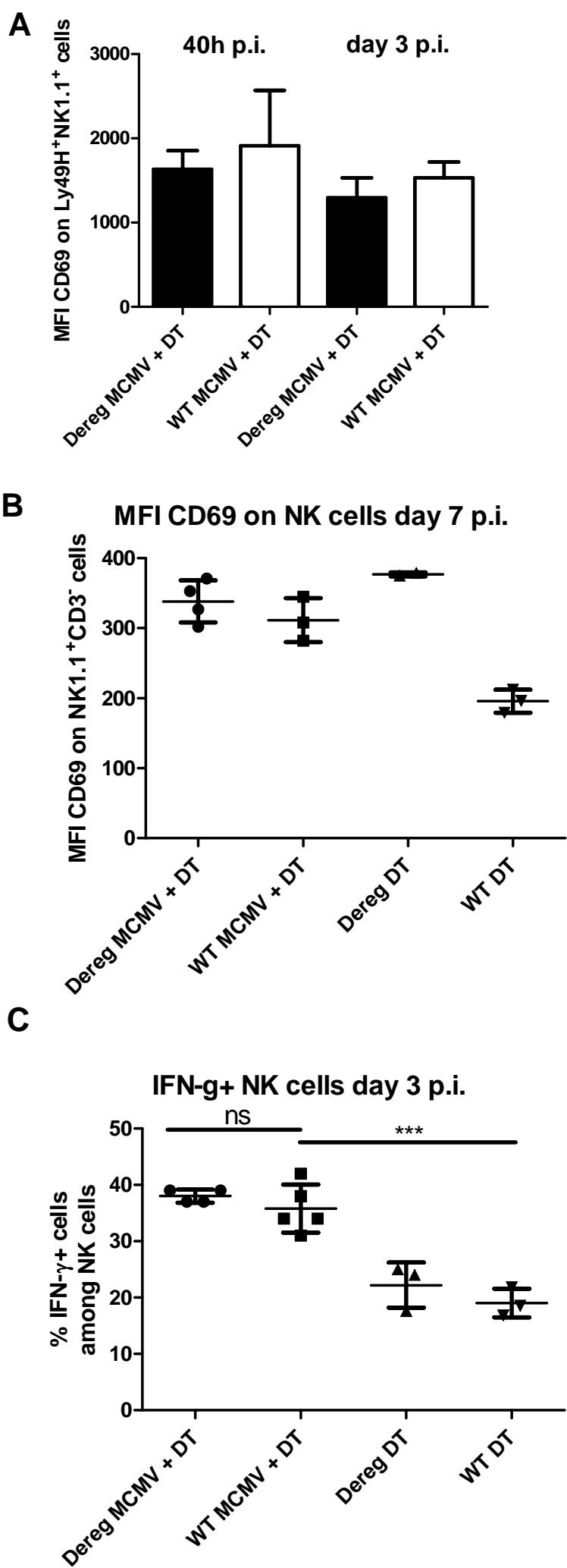

Supplement: Supplementary file 3 — Additional file 3: Figure S3: Activation and IFN-γ production by NK-Ly49H+ cells in Treg depleted mice. (A) Shows activation with regard to CD69 expression on Ly49H+NK1.1+ cells upon infection during Treg depletion. Data shown is representative of at least two experiments with 3-4 mice per group. (B) Expression of early activation marker CD69 on NK cells of infected mice is clearly reduced on day 7 in comparison to 40 h and 3 days p.i. But the results support the observation that Treg depletion under homeostatic conditions leads to higher NK cell activation. Data shown is representative of two experiments with 2-4 mice per group. (C) PMA/Ionomycin restimulation assay for splenic NK cell IFN-γ production showed similar results as IL-2 restimulation but with higher unspecific ex vivo activation. Groups consisted of 3-5 mice and significance was determined by two tailed, unpaired Student’s t test. (***) p < 0,0001; (ns) not significantly different. (PDF 21 KB) [file 12985_2014_2471_MOESM3_ESM.pdf]
